# Therapeutic Effect of Rapamycin-Loaded Small Extracellular Vesicles Derived from Mesenchymal Stem Cells on Experimental Autoimmune Uveitis

**DOI:** 10.3389/fimmu.2022.864956

**Published:** 2022-03-29

**Authors:** Huan Li, Zhihui Zhang, Yongtao Li, Lin Su, Yanan Duan, Hui Zhang, Jinying An, Tianwen Ni, Xiaorong Li, Xiaomin Zhang

**Affiliations:** Tianjin Key Laboratory of Retinal Functions and Diseases, Tianjin Branch of National Clinical Research Center for Ocular Disease, Eye Institute and School of Optometry, Tianjin Medical University Eye Hospital, Tianjin, China

**Keywords:** experimental autoimmune uveitis (EAU), uveitis, mesenchymal stem cells, small extracellular vesicles (sEVs), rapamycin

## Abstract

Autoimmune uveitis is a major cause of vision loss and glucocorticoids are major traditional medications, which may induce serious complications. Rapamycin has been demonstrated to exhibit immunosuppressive effects and is promising to be used in treating uveitis by intravitreal injection. However, repeated and frequent intravitreal injections increase the risk of severe ocular complications, while the efficacy of subconjunctival injection of rapamycin is low since it is difficult for rapamycin to penetrate eyeball. Recently, small extracellular vesicles (sEVs) have attracted considerable research interest as natural drug delivery systems that can efficiently cross tissues and biological membranes. SEVs derived from mesenchymal stem cells (MSC-sEVs) also can exert immunosuppressive effect and ameliorate experimental autoimmune uveitis (EAU). The aim of this study was to construct a Rapamycin-loaded MSC-sEVs delivery system (Rapa-sEVs) and investigate its therapeutic effect on EAU by subconjunctival injection. Rapa-sEVs were prepared by sonication and characterized by nanoparticle tracking analysis, transmission electron microscopy, and western blotting. Clinical and histological scores were obtained to assess the treatment efficacy. Additionally, T cell infiltration was evaluated by flow cytometry. The results indicated that Rapa-sEVs could reach the retinal foci after subconjunctival injection. Compared to sEVs and rapamycin alone, Rapa-sEVs can produce a more marked therapeutic effect and reduce ocular inflammatory cell infiltration. Overall, MSC-sEVs have significant potential for the delivery of rapamycin to treat EAU. Subconjunctival injection of Rapa-sEVs may be contender for efficacious steroid-sparing immunomodulatory therapy.

## Introduction

Autoimmune uveitis is a common inflammatory ocular disease that often causes vision loss or blindness due to macular edema, ocular hypertonia, and retinal ischemia (1). It is an intractable problem in the treatment of uveitis in the chronic recurrent stage. Glucocorticoids (GCs) are traditional medications. However, prolonged use of high-dose GCs may induce systemic side effects, including hypertension, hyperlipidemia, and diabetes (2, 3). Local application of GCs allows a smaller dose and reduces systemic side effects, but patients may develop cataracts, ocular hypertension, or glaucoma (2–4). These adverse reactions limit the clinical use of GCs in uveitis. Thus, there is a pressing need for efficacious steroid-sparing immunomodulatory therapies.

Rapamycin (also known as sirolimus) is a bacteria-derived immunosuppressing agent. It is an inhibitor of mammalian targets of rapamycin (mTOR), which can suppress T-cell proliferation by binding to FK-506-binding protein (FKBP)-12 (5). Therefore, it is thought to be useful for the treatment autoimmune uveitis (6, 7). Unfortunately, systemic administration of rapamycin is associated with adverse effects, including cytotoxicity, particularly in hematologic diseases (8–13). In recent years, some clinical studies have assessed the efficacy and safety of intravitreal rapamycin for the management of uveitis (14–17). Many patients with uveitis have a prolonged course and are vulnerable to recurrence. They may necessitate repeated and frequent intravitreal injections, which can result in several complications such as endophthalmitis, retinal tears or detachment, and vitreous hemorrhage (18). Subconjunctival injection can reduce the aforementioned risks, but this route of drug delivery may not be effective. Douglas et al. found that rapamycin could only be detected in the vitreous humor of horses until 21 days after subconjunctival injection (19). Therefore, the ability of rapamycin to penetrate the intraocular tissues should be enhanced when subconjunctival injection is applied.

In recent years, some studies have attempted to employ drug carriers to improve rapamycin’s ability to penetrate the blood-retinal barrier, thereby enhancing its therapeutic efficacy for ocular posterior segment diseases. These carriers include micelles, liposomes, niosomes, and different polymeric vesicles (20–22). Nonetheless, these carriers are derived from xenobiotic materials and are vulnerable to clearance by the mononuclear phagocyte system. Small extracellular vesicles (sEVs) are biological nanoparticles with a bilipid membrane secreted by different cell types with a diameter under 200 nm, the main components of which are exosomes. They are ideal carriers for drug delivery because of their distinctive advantages, including small particle size, high biocompatibility, biological barrier penetration, and low immunogenicity (23–25). SEVs have already been successfully tested for loading several drugs, such as curcumin (26, 27), paclitaxel (28, 29), and doxorubicin (30, 31). Mesenchymal stem cells (MSCs) are the most prolific producers of sEVs (32). SEVs derived from MSCs (MSC-sEVs) have similar functions as MSCs, including promoting tissue regeneration, inhibiting autoimmune responses, and protecting neuron function. There have been some encouraging therapeutic effects of MSC-sEVs in various animal models. In our previous studies, we showed that MSC-sEVs could ameliorate experimental autoimmune uveitis (EAU) in rats (33). Therefore, they are ideal carriers for drugs targeting the retina and can be used to deliver drugs to treat autoimmune uveitis. In this study, we attempted to formulate Rapamycin-loaded sEVs (Rapa-sEVs) and investigate their effects in treating EAU.

## Materials and Methods

### Animals

Female C57BL/6J mice (6–8 weeks old) with no ocular or systemic diseases were purchased from Vital River (Beijing, China). All protocols involving mice were approved by the Animal Care and Use committee of Tianjin Medical University Eye Hospital and conformed to the ARVO Statement for the Use of Animals in Ophthalmic and Vision Research.

### Culture and Identification of MSCs

Human umbilical cord MSCs were provided by Beijing Beilai Biological Co., Ltd. (China), and MSC isolation and culture were performed as previously described (33). Umbilical cords obtained from normal pregnancies without complications after cesarean section delivery were placed immediately in saline containing penicillin (100U/mL) and streptomycin (100μg/mL) (Gibco, USA), then transported to the laboratory within 6 h. After removing residual blood and the blood vessels, the obtained Wharton’s jelly was cut into 1-3 mm^3^ pieces and digested with 0.1% type-2 collagenase (Gibco) and 0.125% trypsin (Gibco) at 37°C for 1 h. The suspension was then filtered through a 100-mesh screen to remove the undigested tissue. The supernatant from the filtration was centrifuged and washed three times with PBS. The cell precipitate was resuspended in Dulbecco’s modified Eagle’s medium/nutrient mixture F12 (DMEM/F12, Invitrogen, USA) complete medium. The medium contained 10% fetal bovine serum (FBS; Invitrogen), 100 U/ml penicillin, and 100 μg/ml streptomycin. The cells were seeded in a T175 flask and cultured at 37°C in a 5% CO_2_ incubator. The medium was changed every 3 days. When cell fusion reached 80%, the passage was carried out at a subculture ratio of 1:2, and cells from P3 to P5 were used for experiments.

According to the criteria proposed by ISCT (34), flow cytometry was used to identify the surface markers of MSCs, including CD73 (eBioscience, USA), CD90 (eBioscience), CD45 (eBioscience), and CD34 (eBioscience). Meanwhile, the osteogenic and chondrogenic differentiation abilities of MSCs were demonstrated by staining *in vitro*.

### Isolation of MSC-sEVs

To generate exosome-free FBS, FBS was centrifuged overnight (approximately 14 h) at 110,000 × g at 4°C. When cell fusion reached 60%, the cells were cultured in complete medium with 10% exosome-free FBS for 24 h. Then, supernatants were collected and sEVs were isolated by ultracentrifugation at 4°C. Specific steps include 300 × g for 10 min, 2000 × g for 20 min, 10,000 × g for 30 min, and 110,000 × g for 2h, followed by filtration through a 0.22-μm filter. In all experiments, sEVs were used immediately after ultracentrifugation. The BCA protein assay kit (Solarbio, China) was used to measure the total protein content of the sEVs.

### Drug Entrapment Into sEVs

Rapa-sEVs were prepared by sonication. Rapamycin (Sigma-Aldrich, USA) was mixed with sEVs at a 9:1 concentration ratio. The mixture was incubated for 10 min and sonicated using an ultrasonic cell crusher (25% power, 6 cycles of a 30 s pulse/30 s pause). The mixture was incubated at 37°C for 1 h to allow the recovery of the sEV membrane. Ultrafiltration centrifugation was used to remove the free rapamycin.

### Characterization of sEVs and Rapa-sEVs

SEVs and Rapa-sEVs were characterized according to the criteria proposed by members of four societies (SOCRATES, ISEV, ISCT and ISBT) (35). Nanoparticle-tracking analysis (NTA) was used to analyze the size distribution of sEVs and Rapa-sEVs. The particle size was analyzed using NTA software (version 3.3, Nanosight). To further observe the size and morphology of these particles, transmission electron microscopy (TEM) was performed. First, samples were fixed with paraformaldehyde for 5 min and loaded onto Formvar-coated grids. Phosphotungstic acid was used for negative staining. Finally, the samples were dried and observed by TEM at 80.0Kv. Markers on the surface of sEVs and Rapa-sEVs, such as CD9, CD63, and TSG-101, were detected by western blot. Proteins were extracted from normal sEVs (N-sEVs), sonicated sEVs (S-sEVs), and Rapa-sEVs using a cell lysis buffer. The BCA Protein Assay Kit was used to determine the total protein concentrations of the three samples, according to the manufacturer’s recommendations. The samples were then boiled at 95°C for 5 min. 20 μg of protein was electrophoresed and transferred to a polyvinylidene difluoride (PVDF) membrane. Membranes were blocked with 5% non-fat dried milk and incubated with primary antibodies overnight at 4°C. The primary antibodies included antibodies against CD9 (Abcam, UK), CD63 (Abcam), TSG-101 (Abcam), and β-actin (Abcam). The membranes were then incubated with secondary antibodies for 2 h. Protein bands were visualized using Western Lightening Chemiluminescence reagents.

The concentration of rapamycin in Rapa-sEVs was detected by high performance liquid chromatography (HPLC) (Thermo Scientific UltiMate 3000 series). A series of rapamycin at concentrations ranging from 0 to 50 µg/mL were prepared to generate the standard curve. An aliquot of 20 μl of standard solution and sample solution was injected into the HPLC system for analysis. Taking the average peak area as transverse coordinate and the concentration of rapamycin as longitudinal coordinate, the standard curve was drawn and the regression equation was calculated. Acetonitrile was added to Rapa-sEVs to precipitate the proteins of sEVs and extract rapamycin. This solution was centrifuged, and the supernatant was used for HPLC. The chromatographic column was a C18 reverse phase column. For the mobile phase, HPLC-grade acetonitrile and water (Vacetonitrile/Vwater= 65/35) were used at a flow rate of 1ml/min. The detection wavelength was 278 nm, and the column temperature was 62°C. The drug loading capacity was calculated according to the formula: Loading Capacity (%) = (Drug entrapped)/(Drug entrapped + The total mass of sEVs) × 100%. The encapsulation efficiency was calculated as follows: Encapsulation Efficiency (%) = (Drug entrapped)/(Total amount of drug) × 100%.

### Induction of EAU and Treatment Methods

Mice were immunized with a subcutaneous injection of 200 μl emulsion containing 250 μg human interphotoreceptor retinoid binding protein peptide [LAQGAYRTAVDLESLASQLT (hIRBP651-670), Shanghai Hanhong Chemical Co., Ltd., Shanghai, China] and 3.5 μg Mycobacterium tuberculosis (BD Biosciences) emulsified with complete Freund’s adjuvant (CFA, Sigma Aldrich). Pertussis toxin (PTX, List Biological Laboratories Inc.) was administered intraperitoneally at 0.5 μg/dose, and repeated 24 h later. The mice were injected subconjunctivally with sEVs and Rapa-sEVs at day 11 and day 16 following immunization. Approximately 10 µL of suspension (the protein concentration of sEVs was 1μg/μl) was delivered with a 33 gauge needle per injection. Mice in the control group were injected with the same volume of PBS and rapamycin the drug content was same as Rapa-sEV treated group.

### Trace of sEVs and Rapa-sEVs

SEVs were labelled with PKH26 fluorescent dye (PKH26 fluorescent cell linker kit, Sigma-Aldrich). The immunized mice were injected subconjunctivally with sEVs and Rapa-sEVs on day 18 following immunization. The mice were sacrificed after 24 h (n=3) and 48 h (n=3), and the eyes were processed for frozen sections. After staining with DAPI (Sigma-Aldrich), the sections were observed under a confocal microscope.

### Clinical and Histological Assessment

On day 9 post-immunization, the fundi of the mice (each group, n=6) were observed by mydriatic binocular indirect ophthalmoscopy on alternate days. On day 21 post-immunization, the mice (each group, n=6) were sacrificed by cranio-cervical dislocation. Their eyes were collected and embedded in paraffin, sectioned (4μm), and stained with hematoxylin and eosin (HE). Clinical and histological grades were scored according to the criteria reported by Caspi et al (36).

### Optical Coherence Tomography

On day 18 post-immunization, the mice (each group, n=6) were systemically anesthetized. The pupils were anesthetized and dilated with 0.1% tropicamide. The mice were weared using a corneal contact lens. Spectralis optical coherence tomography (OCT) (Heidelberg, Germany) was used to scan the retina. Scores were recorded according to the criteria described by Gadjanski’s group (37).

### Flow Cytometry Analysis

On day 18 post-immunization, eyes and cervical draining lymph nodes of EAU mice (each group, n=3) were collected, and cell suspensions were prepared. The cells were surface stained with anti-CD4 antibody (BioLegend) for 30 min at 4°C. After fixation and permeabilization, the cells were stained with an anti-Foxp3 antibody (BioLegend) to detect Foxp3^+^ cells. For intracellular staining of interferon (IFN) -γ and interleukin (IL)-17, cells were were pretreated for 4 to 6 h with 50 ng/ml photoblog 12-myristate 13w-acetate, 1μg/ml ionomycin, and 1μg/ml brefeldin A (Sigma-Aldrich), and then incubated with antibodies against IFN-γ and IL-17 (BioLegend) after fixation and overnight permeabilization. Data collection was performed on a FACS Calibur flow cytometer (BD Biosciences, USA), and analyzed using flow cytometry software (FlowJo, USA).

### Statistical Analysis

For all experiments, data are presented as mean ± standard deviation (SD). Tests for significance of differences between the groups were performed using the Kruskal-Wallis test or one-way analysis of variance (one-way ANOVA) in GraphPad Prism 8.0 (GraphPad Software, USA). A minimum p value of 0.05 was chosen as the significance level.

## Results

### Identification of MSCs and sEVs

**Figure 1:**
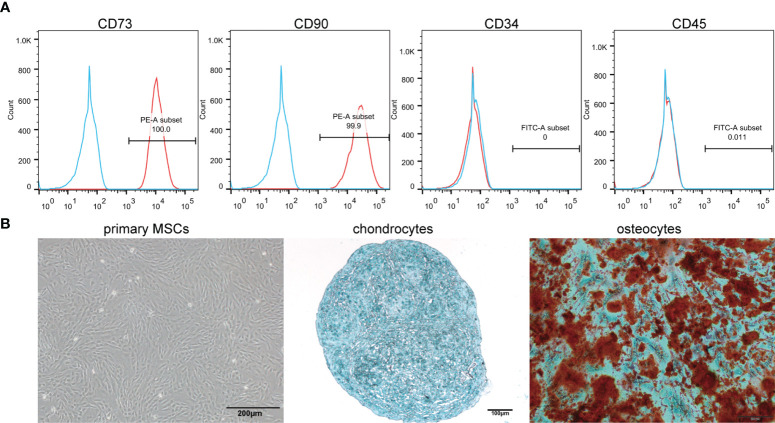
Identification of MSCs. **(A)** Immunophenotypic characterization of hMSCs was performed by flow cytometry. The vast majority of cells (>99%) were positive for CD73 and CD90, but a few cells (<0.1%) expressed CD34 and CD45. **(B)** Primary cells presented as morphologically homogeneous, with elongated spindle appearance. The hMSCs were cultured in appropriate differentiation media for 3 weeks. For chondrogenic differentiation, the fixed chondrospheres were embedded and cut into sections and stained with alcian blue. The induced cells were stained by oil red O to indicate osteocytes.

**Figure 2:**
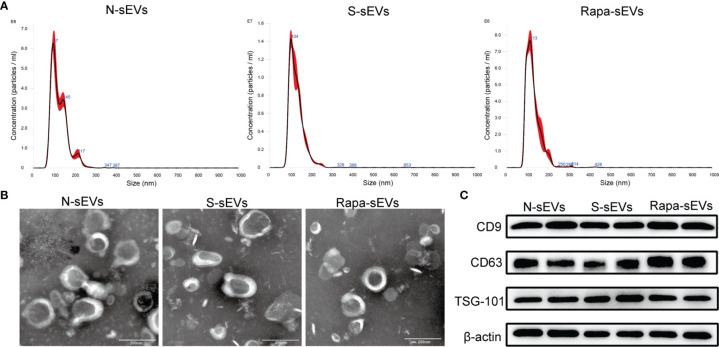
Identification of sEVs. **(A)** NTA displayed that the diameters of most sEVs in each group were around 100nm. **(B)** TEM showed that sEVs in each group were 50–200 nm disc-like vesicles with bilayer membrane. **(C)** Western blot analysis showed that sEVs of all groups express CD9, CD63, and TSG101.

The surface antigens of MSCs were identified by flow cytometry. The expression of CD73 and CD90, and the absence of CD34 and CD45 were confirmed (**Figure 1A**). Under light microscopy, the primary MSCs were plastic-adherent and spindle-shaped (**Figure 1B**). In addition, MSCs were functionally characterized by differentiation into osteogenic and chondrogenic phenotypes in different differentiation media (**Figure 1B**). The sizes of the sEVs were analyzed by NTA. The size distribution of N-sEVs, S-sEVs and Rapa-sEVs ranged from 50 to 200nm (**Figure 2A**). TEM showed that sEVs were cup-shaped in all three groups (**Figure 2B**).

Western blot analysis confirmed that the vesicles expressed sEVs markers including CD63, CD9, and TSG101 (**Figure 2C**), indicating that most of the vesicles were sEVs.

**Figure 3:**
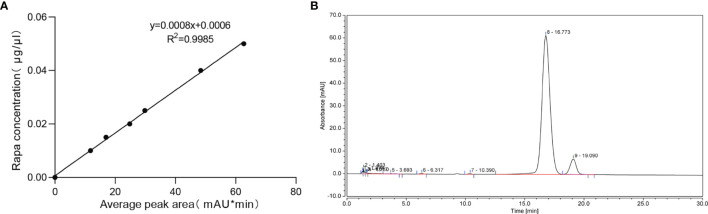
**(A)** The linear regression equation of rapamycin standard curve. **(B)** The chromatographic peak of rapamycin in Rapa-sEVs detected by HPLC.

The drug loading capacity and encapsulation efficiency were analyzed by HPLC. The results showed that a good shape peak could be detected at 16.773 min (**Figure 3B**). The linear regression equation of rapamycin standard curve was Y=0.0008X+0.0006, R^2^ = 0.9985(**Figure 3A**). The drug loading capacity of rapamycin in Rapa-sEVs was 45.7 ± 1.3%, and the encapsulation efficiency was 82.1 ± 4.3%.

### SEVs Penetrated Ocular Wall and Reached the Retina Rapidly

**Figure 4:**
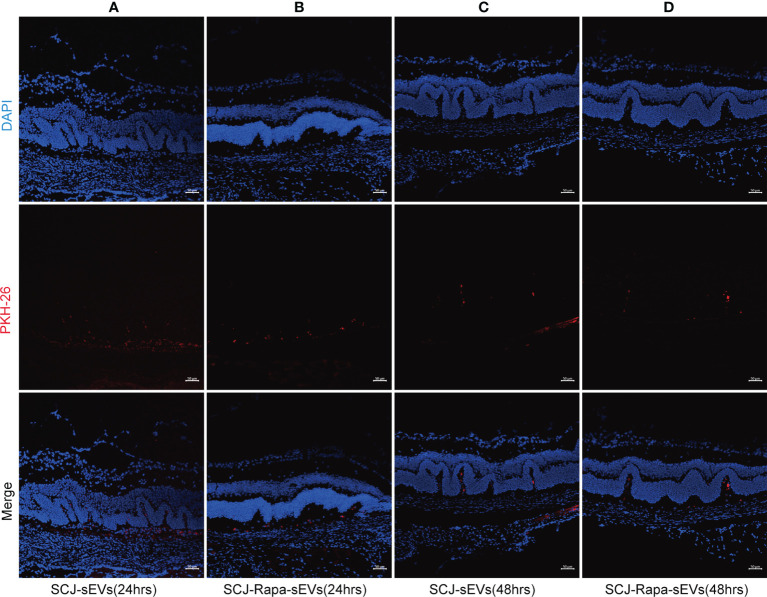
Trace of sEVs. Distribution of sEVs labeled with PKH-26 in the retina was observed at 24 h and 48 h after subconjunctival injection. 24 h after the injection of sEVs **(A)** and Rapa-sEVs **(B)**, fluorescence was detected in the retina. A mild reduction in red fluorescence was observed 48 h after the injection of sEVs **(C)** and Rapa-sEVs **(D)**.

SEVs are the natural drug carriers. The ability of sEVs to deliver drugs to retina is essential for therapeutic efficiency. 24 h after subconjunctival injection of sEVs (**Figure 4A**) and Rapa-sEVs (**Figure 4B**), red fluorescence (PKH-26) was detected in the retina foci by confocal microscopy. It was still visualized 48 h post-injection (**Figures 4C, D**) but decayed compared to 24 h after injection.

### Subconjunctival Injection of Rapa-sEVs Ameliorated Uveitis in Mice

**Figure 5:**
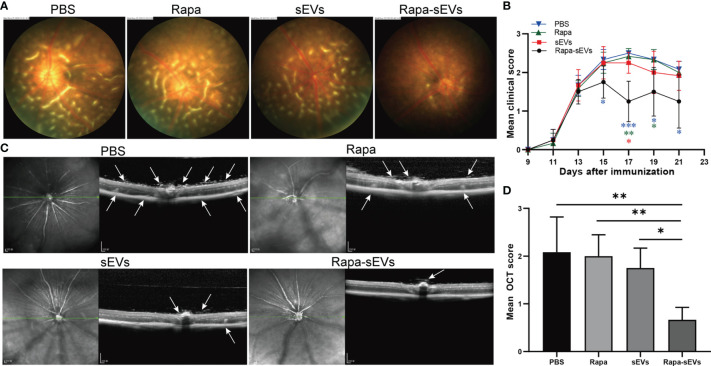
Subconjunctival injection of Rapa-sEVs ameliorated uveitis in mice. Fundus images and OCT at day 18 post-immunization showed ocular inflammation in PBS, Rapa, and sEV treated groups **(A, C)**. In contrast, Rapa-sEV treatment led to decreased ocular inflammation **(A, C)**. **(B)** Clinical observation of EAU mice treated with PBS, Rapa, sEVs, and Rapa-sEVs. ***P < 0.001, **P < 0.01, *P < 0.5, Kruskal-Wallis test **(D)** OCT was performed at day 18 post-immunization, and results are expressed quantitatively as OCT scores. The inflammatory cells in the vitreous, retinal folders, and retinal detachment near the optic disk were indicated by white arrows. Values are expressed as the mean ± SD of six mice (6 eyes) per group. **P < 0.01, *P < 0.5, Kruskal-Wallis test.

**Figure 6:**
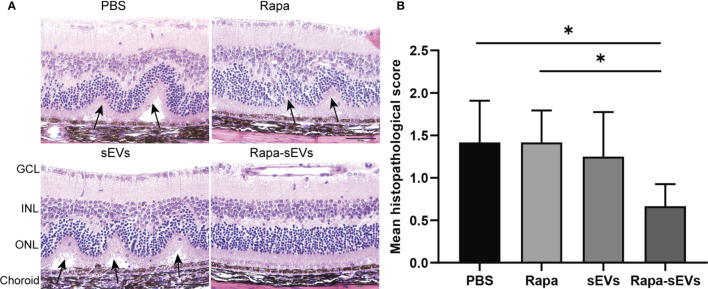
Histological assessment of the retina in EAU. **(A)** Representative H&E staining images of PBS, Rapa, sEV, and Rapa-sEV treated groups. The black arrows represented the retinal folds and detachments near the optic disk. GCL, ganglion cell layer; INL, inner nuclear layer; ONL, outer nuclear layer. **(B)** Results are expressed quantitatively as histopathological scores. Values are expressed as the mean ± SD of six mice (6 eyes) per group. *P < 0.05, Kruskal-Wallis test.

EAU mice were treated twice at day 11 and 16 post immunization. The clinical scores of mice treated with Rapa-sEVs decreased significantly from day 15 compared to those in all other groups (Kruskal-Wallis test) (**Figure 5B**). Representive pictures of the fundus at the peak stage were shown in **Figure 5A**, which demonstrated that multiple linear and large confluent lesions were observed in the fundus of mice in the PBS and Rapa control groups, and some confined lesions were found in the MSC-sEV treated group, while only very few small focal lesions near the optic disk were detected in the fundus of mice treated with Rapa-sEVs. On OCT (**Figure 5C**) performed at the same point as fundus photography, remarkable inflammatory cell infiltration in the vitreous and retinal disorders, including retinal folders and detachment were found in Rapa and PBS groups, while very few inflammatory cells were found in the vitreous and the retinal structure was almost normal in the mice treated with Rapa-sEVs. The scores of OCT changes in Rapa-sEV group were significantly lower than those of the other three groups (Kruskal-Wallis test) (**Figure 5D**). In addition, the histopathological evaluations on day 21 after immunization were in accordance with the OCT findings (Kruskal-Wallis test) (**Figure 6**). Few inflammatory cells infiltrated in the retina and no retinal folders and detachments were found in the Rapa-sEV treated group. These results indicated that subconjunctival injection of Rapa-sEVs attenuated ocular inflammation and protected retinal structure.

### Rapa-sEVs Treatment Reduced Inflammatory Cell Infiltration in the Rye

**Figure 7:**
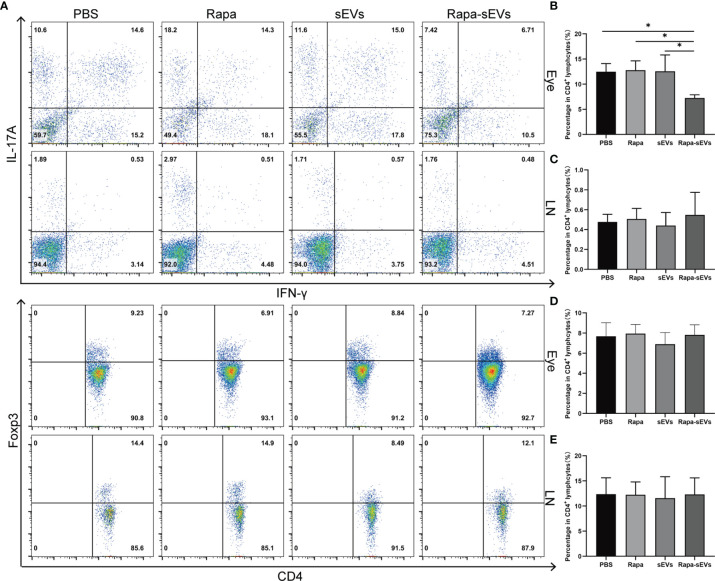
Analysis of T cell subsets from eye and lymph node in EAU. **(A)** Representive figures of intracellular staining of IFN-γ^+^IL-17^+^, Foxp3^+^ in eyes and lymph nodes derived CD4^+^T cells from PBS, Rapa, sEV, and Rapa-sEV treated mice with EAU. **(B)** The percentage of CD4^+^IFN-γ^+^IL-17^+^ cells in eyes. **(C)** The percentage of CD4^+^IFN-γ^+^IL-17A^+^ cells in cervical draining lymph nodes. **(D)** The percentage of CD4^+^Foxp3^+^ cells in eyes. **(E)** The percentage of CD4^+^Foxp3^+^ cells in cervical draining lymph nodes. Values are expressed as the mean ± SD of three mice per group. *P < 0.05, one-way ANOVA.

IFN-γ and IL-17 double‐positive T cells are involved in a variety of autoimmune diseases, such as rheumatoid arthritis (38), multiple sclerosis (39), and inflammatory bowel disease (40). Compared with the classical Th17 cells, CD4^+^IFN-γ^+^IL-17^+^ cells are more pathogenic and are considered as pathogenic T cells in autoimmune response (41).To further explore the therapeutic effects of Rapa-sEVs on retinal inflammation, we assessed the frequency of CD4^+^IFN-γ^+^IL-17^+^, CD4^+^Foxp3^+^cells in the eyes and lymph nodes on day 18 post-immunization by flow cytometry. As shown in **Figure 7**, Rapa-sEV treatment downregulated the proportions of CD4^+^IFN-γ^+^IL-17^+^ cells in the retina (one-way ANOVA) (**Figure 7B**), but did not affect T cells in lymph nodes (one-way ANOVA) (**Figure 7C**). No differences of the percentage of CD4^+^ Foxp3^+^ Treg cells among all groups were found (one-way ANOVA) (**Figure 7D, E**).

## Discussion

In this study, rapamycin was loaded into sEVs by sonication. This formulation was administrated to EAU mice *via* subconjunctival injection at the onset and peak of disease. The results of the present study reveal that Rapa-sEVs can alleviate retinal inflammation and protect the retinal structure of mice with EAU. In conclusion, sEVs play an important role in drug delivery, especially for hydrophobic drugs.

Rapamycin was considered an antifungal agent in the early 1970s. However, the pharmacological potential of rapamycin was not discovered until years later, including immunosuppressive, antiangiogenic, and antiproliferative effects. In contrast to other immunosuppressive agents, including tacrolimus and cyclosporine, rapamycin inhibits mTOR by binding to FKBP-12. This prevents the cell cycle transition from G1 to S phase, inhibits protein translation initiation, and results in antiproliferative effects (5).

It has been demonstrated that rapamycin exhibits therapeutic potential for uveitis in animal experiments and clinical trials. Several preclinical studies have demonstrated the ability of rapamycin to significantly inhibit experimental uveitis (42, 43). In addition, rapamycin showed a synergetic effect with other agents, such as tacrolimus, cyclosporine, and corticosteroids (44–46). A retrospective clinical study evaluated the therapeutic role of oral low-dose rapamycin in active uveitis (47). The results showed that rapamycin might have a limited role in the treatment of severe uveitis (47). Although low-dose rapamycin appeared to be well tolerated, the incidence of adverse effects was high. Topical applications can reduce the systemic side effects. An experimental study using New Zealand white rabbits revealed that intravitreal injection of rapamycin had good safety, tolerance, and stability (48). In another study, rapamycin was detected quickly in the aqueous humor and vitreous of horses with equine recurrent uveitis after intravitreal injection (19). However, until 21 days after subconjunctival injection, rapamycin in the aqueous humor could not be detected (19). This study demonstrated that intravitreal injection may be an efficient route of administration. Intravitreal injection of rapamycin has entered phase III clinical trials. A clinical study, known as SAKURA, was performed to evaluate the therapeutic effects of intravitreal rapamycin (three different doses: 44 μg, 440 μg, and 880 μg) in noninfectious uveitis (17). The results indicated that intravitreal rapamycin 440 μg ameliorated ocular inflammation (17). However, further studies are required to identify its safety and efficacy.

Repeated intravitreal injections may increase the risk of infection and the financial and psychological burden of patients. In the present study, rapamycin was loaded into the sEVs and administrated by subconjunctival injection. As drug carriers, sEVs have many advantages. The lipid bilayers of sEVs can effectively protect loaded drugs from degradation and allow them to remain stable for a long period of time. Moreover, it can provide the advantages of nanotechnology for efficient drug transport capable of overcoming various biological barriers (49), such as the blood-ocular barrier (**Figure 4**). SEVs are naturally occurring membrane vesicles that are secreted by nearly all cell types in all body fluids demonstrating excellent biocompatibility. SEVs have been shown to reduce inflammation in EAU. Therefore, Rapa-sEVs may play a dual role in therapeutic actions. In addition, sEVs may be repeatedly freeze thawed while retaining their original morphology and other characteristics. Finally, sEVs have a low risk of immunogenicity and tumorigenicity compared to cells.

Therapeutic agents can be incorporated into sEVs using different approaches, including incubation, sonication, extrusion, and electroporation. A study investigated two methods: incubation at 37°C and sonication for PTX loading (50). The results revealed that sonication resulted in the greatest loading efficiency (50). Kim et al. compared three methods of loading sEVs with PTX and arrived at the same conclusion (51). In addition, this method does not significantly affect the membrane-bound proteins of sEVs, which is the same as that reported in our study.

However, there are several deficiencies in the present study. Further research should be conducted to investigate in detail the drug release kinetics and drug toxicity of Rapa-sEVs. In conclusion, sEVs as delivery vehicles are attractive and promising for autoimmune uveitis. Moreover, to promote its clinical transformation, further investigations regarding the potency and toxicology of Rapa-sEVs are necessary.

## Data Availability Statement

The original contributions presented in the study are included in the article/supplementary material. Further inquiries can be directed to the corresponding author.

## Ethics Statement

The animal study was reviewed and approved by the Animal Care and Use committee of Tianjin Medical University Eye Hospital.

## Author Contributions

XZ and HL designed the research and interpreted data. HL, ZZ and YL performed experiments and analyzed data. LS, YD, HZ, JA and TN provided methodological guidance. HL wrote the manuscript. XZ reviewed and approved the final manuscript. XZ and XL provided financial and administrative support. All authors contributed to the article and approved the submitted version.

## Funding

This work was supported by National Natural Science Foundation of China (81870651, 82171042). Tianjin Science and Technology Support Plan (20YFZCSY00990), Natural Science Foundation of Tianjin (20JCZDJC00100) and Tianjin Key Medical Discipline (Specialty) Construction Project.

## Conflict of Interest

The authors declare that the research was conducted in the absence of any commercial or financial relationships that could be construed as a potential conflict of interest.

## Publisher’s Note

All claims expressed in this article are solely those of the authors and do not necessarily represent those of their affiliated organizations, or those of the publisher, the editors and the reviewers. Any product that may be evaluated in this article, or claim that may be made by its manufacturer, is not guaranteed or endorsed by the publisher.
